# A tumor association to be aware: endometrial cancer and colon cancer in relation to Lynch syndrome

**DOI:** 10.1259/bjrcr.20210230

**Published:** 2022-01-12

**Authors:** Behyamet Onka, Daoud ali Mohamed, Romeo Thierry Tessi Yehouenou, Boris Adeyemi, Wend-Yam Mohammed Traore, Mbina Mbougou Kevin arthur, Hounayda Jerguigue, Rachida Latib, Youssef Omor

**Affiliations:** 1 Department of Radiology, National Institute of Oncology, University Hospital Center IBN SINA, Mohamed V University, Faculty of Medicine, Rabat, Morocco; 2 Department of emergency, University Hospital Center IBN SINA, Mohamed V University, Faculty of Medicine, Rabat, Morocco

## Abstract

lynch syndrome (LS) is an autosomal dominant genetic disorder with incomplete penetration caused by a germline mutation in one of the genes of the deoxyribonucleic acid (DNA) mismatch repair system (MMR) namely: mutL homolog 1 (MLH1), mutS homolog 2 (MSH2), mutS homolog 6 (MHS6), post-meiotic segregation increased 1 homolog 2 (PMS2) or the EpCAM (Epithelial CellAdhesionMolecule) gene, which causes the inactivation of MSH2. Patients with this syndrome have a high relative risk of developing cancers at a young age, led by colorectal cancer (CRC) and endometrial cancer in females. The diagnosis is suspected when the patient’s personal and family history meets the Amsterdam or Bethesda criteria. It is guided by immunohistochemistry (IHC) and/or molecular biology that show loss of expression of one or more proteins of the MMR system and microsatellite instability on tumor DNA. In case of positive IHC and/or molecular biology, the patient should be referred to an oncogenetic consultation for a definitive diagnosis. We present the case of a 49-year-old patient who presented an anamic syndrome in metrorrhagia. After a clinical, imaging, biological and anatomopathological examination, the diagnosis of LS was made.

## Clinical presentation

A 49-year-old female who has had moderate bleeding for a few days associated with asthenia and dizziness. She is postmenopausal (has not had her period for five years), married, and has three healthy children. The physical exam found blood stained gynecological wipes, the remainder of the exam was normal. The hemoglobin level was 10 g / dl (reference value 12–16 g / dl).

## Differential diagnosis

Faced with metrorrhagia in a postmenopausal context, we first evoked the diagnosis of endometrial cancer, but a uterine myoma or cervical pathology could not be ruled out.

## Imaging findings, treatment, and follow-up

**Figure 1.**
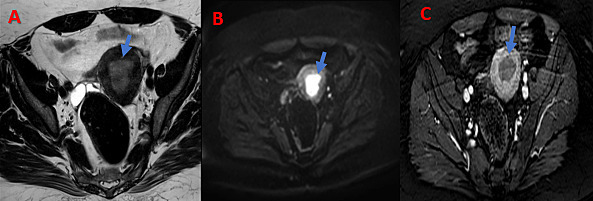
Pelvic MRI in the axial section of the T2 (**A**), diffusion (**B**) and T1 sequence after gadolinium (**C**) showing thickening of the tumor of the endometrium (blue arrow).

Abdominal ultrasound showed an endometrial thickening of 23 mm. Complementary pelvic magnetic resonance imaging (MRI) showed a thickening of the T2 hyper signal tumor of 31 mm with diffusion restriction and enhancement after gadolinium injection (Figure 1).

**Figure 2.**
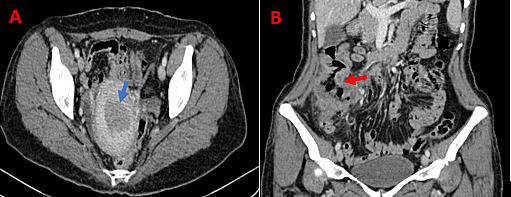
Abdominopelvic CT scan after contrast injection in axial section (**A**) showing thickening of the endometrium (blue arrow) and in coronal section (**B**) showing parietal thickening of the right colon (red arrow).

**Figure 3.**
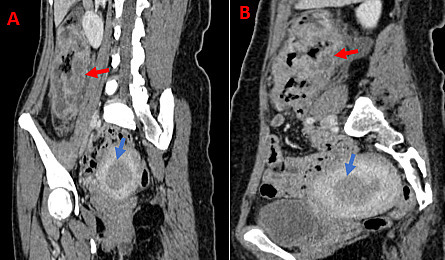
Abdominopelvic CT scan after contrast injection in coronal and sagittal oblique sections (**A and B**) showing thickening of the endometrium (blue arrow) associated with thickening of the right colon (red arrow)

The patient underwent a hysteroscopy endometrial biopsy that was consistent with endometrial adenocarcinoma (ADK). A thoracic-abdominal-pelvic CT scan with injection of contrast medium for extension assessment revealed thickening of the endometrium associated with irregular thickening of the right colon with adjacent adenopathy (Figures 2 and 3). The patient underwent a colonoscopy with biopsy of the right colon, which returned in favor of moderately differentiated colonic adenocarcinoma. The IHC study on colon biopsy showed loss of MLH1 and PMS2 protein expression. The patient underwent a right colectomy and a total hysterectomy at the same time. The postoperative course was simple. The anatomopathological examination of the operative specimens was in favor of a FIGO II Grade 2 endometriotic adenocarcinoma of the World Health Organization (WHO) and a moderately differentiated colonic adenocarcinoma classified PT4bN1a of the American Joint Committee on Cancer (AJCC). We tested for the BRAF mutation (V600E) and a hypermethylation test on the right colectomy specimen, which were negative. Faced with these clinical, imagery, biological, and anatomopathological aspects, we concluded that there was LS.

## Discussion

LS is a genetic predisposition to cancer described in 1913 by Dr. Whartin and then in 1966 by Dr Lynch, who proposed the first diagnostic criteria.^1,2^

The MMR system (which comprises the MLH1, MSH2, MSH6, PMS2 genes) is responsible for the integrity of cell division during replication of repeated DNA sequences called microsatellites. LS reflects a constitutional mutation of one of the genes of the MMR system or of the EPCAM gene (which inactivates MSH2) that causes the loss of its function leading to the development of cancer characterized by a tumor phenotype of microsatellite instability (MSI).^3,4^

LS is a rare autosomal dominant transmission condition with incomplete penetrance varying between 80 and 85%. Its prevalence is estimated between 1/2000 and 1/1000. The mutation is found in 80% of patients and is distributed as follows: 80 to 90% affect the MLH1 and MSH2 genes, 10 to 20% the MSH6 and PMS2 genes, and 3% is linked to a deletion of the 3' end of the EPCAM gene. Other times, LS was called hereditary nonpolyposis colorectal cancer (HNPCC), but the two entities are currently separated. The term LS is reserved for patients with a genetically confirmed mutation, whereas HNPCC is for patients who meet the Amsterdam criteria without a proven genetic mutation. There is also a group of patients with abnormalities of the MMR system without identified mutations: these are the LS-LIKE.^5^

Several cancers are associated with LS, divided into two categories: narrow- and broad-spectrum cancers (Table 1). Cancer risk varies from one study to another and according to the type of mutation. The risk of the MLH1 or MSH2 mutation is 40–70%, while it is 20–50% for the MSH6 or PMS2 mutation. The MSH2 mutation is associated with a higher risk of extradigestive cancers.^6,7^

**Table 1.** Lynch syndrome spectra and cumulative cancer risk

	Type of cancer	Cumulative cancer risk (%)
**Narrow spectrum**	ADK CRC ADK of the endometrium ADK of hail Carcinoma of the upper urinary tract	20–70 10–70 1–10 1–20
**Broad spectrum**	ADK of the stomach ADK of the ovary ADK of the bile ducts Gliobalastoma Sebaceous carcinoma	1–15 2–20 1–3

LS causes 3% of CRC and 2% of endometrial cancers. For CRC the risk is 20–70%, they occur at a young age (50 years), often in the right colon and cecum, with a better prognosis than sporadic CRC and rarely metastatic. For endometrial adenocarcinoma, the risk is approximately 10–70%, also occurring at a young age (50 years). LS-related endometrial cancer is often referred to as “sentinel”, as it is the first cancer to occur in more than 50% of cases.^8–10^

The diagnosis of LS was based in the 1990s on the Amsterdam I and later II criteria (Table 2). However, these criteria are too restrictive with a high false-negative rate. Thus, only 40% of patients with an MMR mutation meet the Amsterdam criteria, and 50–60% of patients with the Amsterdam criteria have a mutation. Faced with the lack of sensitivity and despite the fairly good specificity of the Amsterdam II criteria, the Bethesda criteria revised in 2004 (Table 3) appeared, which are more sensitive but less specific with a high false-positive rate. Several patients with diagnosed LS have been shown to do not meet any of these criteria.^8,11,12^

**Table 2.** Amsterdam II criteria

Patients with the following four criteria:
✓ At least three subjects with cancer on the narrow HNPCC spectrum, one of whom is related to the other two in the first degree
✓ At least one cancer diagnosed before the age of 50
✓ At least two successive generations involved
✓ Exclusion of familial polyposis

**Table 3.** Revised Bethesda criteria

Patients with at least one of the following criteria:
✓ Patient with CRC diagnosed before the age of 50
✓ CRC patient with microsatellite instability and/or loss of MMR protein expression on IHC before age 60
✓ Patient with two synchronous or metachronous cancers belonging to the broad HNPCC spectrum regardless of age
✓ Patient with CRC and two or more first or second degree relatives with cancer of the broad HNPCC spectrum regardless of age
✓ Patient with CRC and a first degree relative with HNPCC broad spectrum cancer diagnosed before 50 years

Clinical criteria are used only to suspect the syndrome and to guide the search for the MSI phenotype. The MSI phenotype is sought on tumor DNA by two techniques. IHC with a sensitivity of 92% and a specificity of 89% finds the loss of expression of two MMR proteins (MLH1/PMS2 or MSH2/MSH6). The second technique for the MSI by polymerase chain reaction (PCR) with a sensitivity of 97% and a specificity of 83%. PCR analyzes a pentaplex of five microsatellite markers. MSI occurs when at least three of the five markers are unstable. It is recommended to use both techniques in cases of suspected LS because if they are normal, this eliminates the diagnosis with high certainty.^8,13,14^

A key notion is to know that an MSI phenotype is not synonymous with LS. In sporadic CRC and endometrial cancer, 80–85% and 15–20%, respectively, are due to hypermethylation of the MLH1 gene and not to a constitutional mutation. In case of cancer with loss of MLH1 expression, it is necessary to look for a BRAF/V600 mutation and hypermethylation of tumor DNA by molecular biology. In case of a positive BRAF mutation and hypermethylation, it is a sporadic cancer and not an LS, and conversely the absence of the BRAF mutation and hypermethylation points to an LS.^15–17^

The certainty diagnosis is based on direct evidence of a mutation in one of the MMR system genes by oncogenetic study. Only patients with MSI tumors after ruling out a BRAF mutation and hypermethylation of MSH1 should have genetic analysis. The percentage of mutation identification is variable: 90% if MSH2 or MSH6 is lost, 70% if the Amsterdam II criteria are met, 40% if MLH1 is lost, 30% if the revised Bethesda criteria are met.^8^

The curative treatment of cancers associated with LS is identical to that of sporadic cancers. Preventive treatment and surveillance have long been debated by several learned societies. There are European and North American management recommendations based on expert consensus. In the case of LS, it is recommended that a colonoscopy be performed every one to two years from the age of 25 onward due to the high risk of CRC. Endometrial cancer is the second most common cancer after CRC and close monitoring by gynecological examination, endovaginal ultrasound, and aspiration biopsy from the age of 35–40 years is recommended, although its benefit is yet to be demonstrated.^12,18^

## Learning points

LS is a rare autosomal dominant genetic disease of incomplete penetrance due to a mutation in the MMR gene. It is characterised by the early occurrence of several familial cancers, notably colorectal and endometrial cancers in the foreground. The lack of recognition of this syndrome leads to delays in the diagnosis of family cancers.

It should be suspected when the Amsterdam II and/or revised Bethesda criteria are met to investigate MSI status and then refer the patient for oncogenetic consultation. Its management should always be discussed in multidisciplinary consultation.
